# Impaired TRPM3-dependent calcium influx and restoration using Naltrexone in natural killer cells of myalgic encephalomyelitis/chronic fatigue syndrome patients

**DOI:** 10.1186/s12967-022-03297-8

**Published:** 2022-02-16

**Authors:** Natalie Eaton-Fitch, Stanley Du Preez, Hélène Cabanas, Katsuhiko Muraki, Donald Staines, Sonya Marshall-Gradisnik

**Affiliations:** 1 School of Pharmacy and Medical Sciences, Griffith University, Gold Coast, Australia; 2 National Centre for Neuroimmunology and Emerging Diseases, Menzies Health Institute Queensland, Griffith University, Gold Coast, Australia; 3 Consortium Health International for Myalgic Encephalomyelitis, Griffith University, Gold Coast, Australia; 4 Université de Paris, INSERM U944 and CNRS UMR 7212, Institut de Recherche Saint Louis, Hôpital Saint Louis, APHP, 75010 Paris, France; 5 Laboratory of Cellular Pharmacology, School of Pharmacy, Aichi-Gakuin University, Nagoya, Japan

**Keywords:** Myalgic encephalomyelitis, Chronic fatigue syndrome, Natural killer cells, Transient receptor potential melastatin 3, Calcium, Naltrexone

## Abstract

**Background:**

Myalgic encephalomyelitis/chronic fatigue syndrome (ME/CFS) is a serious disorder of unknown aetiology. While the pathomechanism of ME/CFS remains elusive, reduced natural killer (NK) cell cytotoxic function is a consistent immunological feature. NK cell effector functions rely on long-term sustained calcium (Ca^2+^) influx. In recent years evidence of transient receptor potential melastatin 3 (TRPM3) dysfunction supports the hypothesis that ME/CFS is potentially an ion channel disorder. Specifically, reports of single nucleotide polymorphisms, low surface expression and impaired function of TRPM3 have been reported in NK cells of ME/CFS patients. It has been reported that mu (µ)-opioid receptor (µOR) agonists, known collectively as opioids, inhibit TRPM3. Naltrexone hydrochloride (NTX), a µOR antagonist, negates the inhibitory action of µOR on TRPM3 function. Importantly, it has recently been reported that NTX restores impaired TRPM3 function in NK cells of ME/CFS patients.

**Methods:**

Live cell immunofluorescent imaging was used to measure TRPM3-dependent Ca^2+^ influx in NK cells isolated from n = 10 ME/CFS patients and n = 10 age- and sex-matched healthy controls (HC) following modulation with TRPM3-agonist, pregnenolone sulfate (PregS) and TRPM3-antaognist, ononetin. The effect of overnight (24 h) NTX in vitro treatment on TRPM3-dependent Ca^2+^ influx was determined.

**Results:**

The amplitude (p < 0.0001) and half-time of Ca^2+^ response (p < 0.0001) was significantly reduced at baseline in NK cells of ME/CFS patients compared with HC. Overnight treatment of NK cells with NTX significantly improved TRPM3-dependent Ca^2+^ influx in ME/CFS patients. Specifically, there was no significance between HC and ME/CFS patients for half-time response, and the amplitude of Ca^2+^ influx was significantly increased in ME/CFS patients (p < 0.0001).

**Conclusion:**

TRPM3-dependent Ca^2+^ influx was restored in ME/CFS patients following overnight treatment of isolated NK cells with NTX in vitro. Collectively, these findings validate that TRPM3 loss of function results in altered Ca^2+^ influx supporting the growing evidence that ME/CFS is a TRP ion channel disorder and that NTX provides a potential therapeutic intervention for ME/CFS.

**Supplementary Information:**

The online version contains supplementary material available at 10.1186/s12967-022-03297-8.

## Background

Transient receptor potential (TRP) channels are a superfamily of polymodal sensors present in many tissue and cell types, and their involvement in sensory reception is well known [1]. The TRPM3 (melastatin) channel acts as a non-selective cation channel that possesses high permeability for calcium (Ca^2+^) [2, 3]. The activation of TRPM3 channels leads to a cascade of intracellular pathways to facilitate cell function in both excitable and non-excitable cells [4]. TRPM3 shares the typical structure of all TRP channels with six transmembrane domains with both the N-terminal and C-terminal domains located within the cytosol. The endogenous neurosteroid, pregnenolone sulfate (PregS) (EC_50_ = 12–32 µM) is commonly used in research to activate TRPM3 ion channel activity to induce an increase in intracellular Ca^2+^ concentration [5] while ononetin (IC_50_ = 0.2–2 µM) rapidly and reversibly inhibits PregS-evoked ionic currents [6]. Changes in the function or activation of TRPM3 would result in changes to Ca^2+^ and as a consequence impacts cell function.

Ca^2+^ is a versatile, universal secondary messenger that facilitates vital biological processes in all cell types. Ca^2+^ ions are in part responsible for promoting intracellular signalling pathways, cell differentiation and proliferation, programmed cell death and transcriptional events in many cell types [7]. The intracellular concentration of Ca^2+^ ([Ca^2+^]_I_) is tightly controlled in homeostatic conditions. Growing evidence suggests that the expression of TRPM3 on natural killer (NK) cells indicates a role of TRPM3 in the regulation of Ca^2+^ in immune cells [8–11]. In addition to the processes described above, the influx of Ca^2+^ in NK cells is important for microtubule rearrangement resulting in granule polarisation, formation of the immune synapse, release of cytolytic granules and granzyme dependent target cell death [12, 13]. Therefore, disturbances in Ca^2+^ homeostasis in lymphocytes can adversely impact immune cell functions and consequentially lead to immune diseases and immunodeficiencies [14].

Myalgic encephalomyelitis/chronic fatigue syndrome (ME/CFS) is a severe multisystemic illness hallmarked by post-exertional neuroimmune exhaustion accompanied by a range of symptoms that are broadly categorised as neurological, immunological, cardiovascular, gastrointestinal and endocrinological [15, 16]. The pathomechanism underlying ME/CFS is unknown, however immunological disturbances have been well reported with emphasis on NK cell dysfunction [17]. In 2016, five single nucleotide polymorphisms (SNPs) were identified in the *TRPM3* gene (rs6560200, rs1106948, rs12350232, rs11142822, rs1891301) in NK cells from ME/CFS patient [18]. Subsequently, flow cytometry revealed a significant reduction in surface expression of TRPM3 and preliminary data reported significantly reduced Ca^2+^ mobilisation in NK cells of ME/CFS patients compared with healthy controls (HC) [8, 9]. Electrophysiological experiments have demonstrated a loss of TRPM3 ion channel function in NK cells of ME/CFS patients compared with HC [10, 11]. Thus, there is a growing body of evidence suggesting the pathomechanism of ME/CFS may be a channelopathy [8, 10, 19]. Therefore, a working hypothesis for the pathophysiology of ME/CFS is that impaired TRPM3 ion channel function limits the influx of Ca^2+^ and downstream signalling pathways, and consequentially impedes cell function.

Currently, no universal treatment exists for ME/CFS, instead treatment is aimed at alleviating targeted symptoms by administering central nervous system (CNS) stimulants, sleeping medications and analgesics [20]. Importantly, TRPM3 has been identified as a thermosensitive and nociceptive channel implicated in detecting pain and heat produced during inflammation with emphasis on heat hyperalgesia and general pain transmission in the CNS [21]. Moreover, the wide spread expression of TRPM3 in areas of the CNS including the cerebrum, brain stem, hypothalamus and hippocampus suggests a role in muscle coordination, cognitive behaviour and memory [8, 22]. These functional properties overlap considerably with symptoms of ME/CFS as epidemiological data has shown ME/CFS patients experience sensitivity for pain with up to 94% of patients reporting muscle aches and pains while 84% report multi-joint pain [23]. Therefore, TRPM3 dysfunction provides a potential therapeutic target for the treatment of ME/CFS.

Recent research has found that naltrexone (NTX) at low doses (LDN) improves the symptoms of ME/CFS [24]. Electrophysiology experiments in ME/CFS patients reported a potential benefit in restoring impaired TRPM3 ion channel activity following treatment of isolated NK cells with NTX [25]. Interestingly, NTX is a long-lasting antagonist to a subfamily of receptors known as opioid receptors (OR) used to treat opioid and alcohol dependence [26]. Mu-OR (µOR) belong to a large and diverse group of membrane receptors known as G protein coupled receptors (GPCRs). GPCRs are widely distributed in the human body where activated G proteins can interact and regulate many effectors or molecules such as Ca^2+^ sensors, ion channels and protein kinases [27]. The activity of TRPM3 can be inhibited by the activation of G protein subunits and direct binding of these subunits to the channel [28]. NTX specifically inhibits the µOR, thus negating these inhibitory effects on TRPM3 [28–30]. Whole-cell patch clamp electrophysiology has been used to investigate the function of TRPM3 ion channels in ME/CFS patients who regularly administered LDN. TRPM3 function yielded similar results between ME/CFS patients taking LDN and HC [31]. As NTX restored TRPM3 ion channel function, it may in turn re-establish Ca^2+^ influx in NK cells leading to normalised downstream signalling pathways and immune functions.

While the function of TRPM3 ion channels have been determined in NK cells of ME/CFS patients using the whole-cell patch clamp technique, this current investigation was designed as a complementary study to evaluate Ca^2+^ influx in order to validate the loss of TRPM3 channel function using an additional method to ensure full potential of research. We aimed, for the first time, to investigate the speed and maximum response of TRPM3-dependent Ca^2+^ influx in ME/CFS patients compared with HC using an immunofluorescent technique. This current investigation provides further insights of the potential therapeutic role of NTX by examining the effect of overnight in vitro treatment on TRPM3-dependent Ca^2+^ influx.

## Methods

### Recruitment

ME/CFS patients and HC were contacted using the National Centre for Neuroimmunology and Emerging Diseases (NCNED) patient database. Participants were screened in accordance to the Canadian Consensus Criteria (CCC) and International Consensus Criteria (ICC) case definitions for ME/CFS using a comprehensive online questionnaire. ME/CFS patients were included if they met the CCC or ICC case definitions for diagnosis and reported being diagnosed by a physician. Potential eligible participants were invited to volunteer in this project. Of those contacted using the NCNED database, 10 ME/CFS patients from South-East Queensland were invited to volunteer in this project. ME/CFS patients were age- and sex-matched with HC. The HC group was defined as those who have not been diagnosed with any underlying illness and are non-fatigued. All participants were aged between 18 and 60 years, had a BMI between 18.5 and 29.9 (kg/m^2^) and were non-smokers.

Participants were excluded if they reported a history of alcohol abuse, cardiovascular disease, diabetes, metabolic syndrome, thyroid disease, malignancies, insomnia, chronic fatigue, and if they were pregnant or breastfeeding. Furthermore, all participants were excluded if they reported the use of pharmacological agents that directly or indirectly interfere with TRPM3 ion channel function as well as Ca^2+^ signalling and immune cell activity. This investigation was approved by the Gold Coast Human Research Ethics Committee (HREC/2019/QGC/56469) and Griffith University Human Research Ethics Committee (GU/2019/1005).

### Participant data collection and sample collection

All participants completed an online questionnaire to provide sociodemographic background, medical history, medications, and symptom history for ME/CFS patients. The 36-item short form health survey (SF-36) and World Health Organization (WHO) Disability Assessment Schedule (DAS) were used to determine level of disability and quality of life (QoL) [32, 33].

Between 7:00 a.m. and 11:00 a.m. at collection locations including Griffith University, Royal Brisbane and Women’s Hospital, Robina Hospital, Toowoomba Base Hospital, Sunshine Coast University Hospital and Tweed Hospital, a total of 84 ml of whole, non-fasted blood was collected from consenting participants into ethylenediaminetetraacetic acid (EDTA) tubes via venepuncture by a qualified phlebotomist. Four ml of EDTA whole blood was used for red blood cell count, white blood cell count and granulocyte cell count within four hours of blood collection for each participant.

### Peripheral blood mononuclear cell and natural killer cell isolations

Samples were deidentified using a unique code and delivered to the laboratory. Eighty ml of blood was used for peripheral blood mononuclear cells (PBMC) isolation by density gradient centrifugation using Ficoll (GE Healthcare, Uppsala, Sweden) as previously described [34]. PBMCs were stained with trypan blue (Invitrogen, Carlsbad, CA, USA) to determine cell count and cell viability. PBMCs were adjusted to a final concentration of 5 × 10^7^ cells/ml for NK cell isolation.

NK cells were isolated by immunomagnetic selection using the EasySep Negative Human NK cell Enrichment Kit (Stem Cell Technologies, Vancouver, BC, Canada). Approximately 2.5–4 × 10^6^ cells NK cells were isolated and used for Ca^2+^ imaging experiments. NK cell purity was defined by CD3^−^CD56^+^ surface expression using flow cytometry (Additional file 1: Figure S1). Specifically, NK cells were incubated for 20 min at room temperature in the presence of CD3 PE-Cy7 (5 µl/test) and CD56 APC (20 µl/test) monoclonal antibodies (Becton Dickinson [BD] Biosciences, San Jose, CA, USA). Cells were acquired at 10,000 events using the Accuri C6 flow cytometer (BD Biosciences, San Diego, CA, USA). The average NK cell purity (%) for this study was 86.73 ± 9.71 (Additional file 1: Figure S2).

### Interleukin-2 stimulation and in vitro drug treatment

Freshly isolated NK cells (~ 4.5 × 10^6^ cells) were stimulated with 20 IU/ml of recombinant human IL-2 (Miltenyi Biotech, BG, Germany) and treated with 200uM NTX (Sigma-Aldrich, St. Louis, MO, USA) for 24 h at 37 °C with 5% CO_2_ in Roswell Park Memorial Institute Medium (RPMI)-1640 (Invitrogen Life Technologies, Carlsbad, CA, USA) supplemented with 10% fetal bovine serum (FBS) (Invitrogen Life Technologies, Carlsbad, CA, USA). The use of IL-2 is designed to support the culture of NK cells overnight.

### Calcium imaging

NK cells (2.0 × 10^5^ NK cells/well) were immobilised using Corning ® Cell-Tak ™ Cell and Tissue Adhesive (BD Biosciences, San Jose, CA, USA) coated 24 well µ-plate (Ibidi, Lochhamer Schlag, Germany). NK cells were incubated for 30 min at 37 °C with 1 µM Fluo-8 (Abcam, Cambridge, UK) with 0.02% Pluronic F127 (Thermofisher, Waltham, Massachusetts, United States). Cells were then washed in indicator free solution and incubated for a further 30 min at room temperature to allow for de-esterification of Fuo-8 AM. All experiments were performed at room temperature (23 ± 2 °C) and in a 1.8 mM Ca^2+^ solution. The Ca^2+^ solution was prepared in milliQ water and contained: NaCl 140 mM, KCl 5.4 mM, CaCl_2_ 1.8 mM. MgCl_2_ 1.0 mM, HEPES 10 mM, NaOH was used to adjust pH to 7.4 and osmolality was adjusted to 300 mOsm/L using D-Glucose.

NK cells were imaged using the Nikon A1R microscope, and fluorescence emissions were monitored using a iXon Life 888 Electron Multiplying CCD camera. Baseline Ca^2+^ was imaged for 2 min before cells were stimulated with 50 µM PregS followed with 1 µM ionomycin for 3 min each. Alternatively, due to the natural decline in fluorescence following peak PregS-dependent Ca^2+^ flux, the effect of ononetin was measured using a separate protocol. In order to desensitize TRPM3 channels, baseline Ca^2+^ was imaged for 3 min in the presence of 4 µM of ononetin followed by 4 µM ononetin + 50 µM PregS, followed by 50 µM PregS and lastly 1 µM ionomycin. Ca^2+^ flux measurements in response to ononetin was added as supplementary material to demonstrate effective inhibition of 50 µM of PregS (Additional file 1: Figure S3). The use of 1 μM of ionomycin aligns with previous literature [9, 35, 36]. The concentration of PregS and ononetin was determined using dose response analysis (Additional file 1: Figure S4).

Variations in fluorescence were measured using NIS Research Elements (Nikon, NIS-Elements V5.2, Tokyo, Japan). Three measurements were reported to represent Ca^2+^ influx. Amplitude value represents the peak of Ca^2+^ influx curve upon activation by PregS and from this the half-time of maximum (T1/2) response was also determined. The rate of Ca^2+^ influx was determined using the initial slope of the curve as calculated using OriginLabs. PregS dependent Ca^2+^ influx measurements were normalised against ionomycin response curves to give the proportion of maximum response.

### Chemicals and reagents

PregS (product code: RDS537650) and ononetin (produce code: RDS514350) were purchased from In Vitro Technologies and stock solutions were prepared at 100 mM in 100% DMSO and stored according to the suppliers’ instructions. NTX (product code: N3136-100MG) was prepared fresh prior to each experiment and reconstituted at 100 mM in distilled water. IL-2 was purchased from Miltenyi Biotec (product code: 130-097-744) stored at 100,000 IU stock in distilled water for up to 1 month. Ionomycin was purchased from Sigma Aldrich (product code: I9657-1MG) and resuspended at 10 mg/ml in 100% DMSO and stored for up to 1-month at − 20 °C. Fluo-8 was purchased from abcam (product code: ab142773) and stored at 1 mM aliquots for up at one month in 100% DMSO. Flow cytometric antibodies were purchased from BD Biosciences, CD3 PE-Cy7 (product code: 563423) and CD56 APC (product code: 555518).

### Statistical analysis

Shapiro–Wilk test was used to assess normality of distribution of investigated parameters. Additional visual observation of histogram plots was completed. Data presented as mean ± standard deviation (SD) unless otherwise stated. Differences were tested using the Mann–Whitney U non-parametric T test. Flow cytometry data were exported from Accuri C6 software. Fluorescence and time-course data were exported from NIS-Elements Advanced Research version 5.2. Statistical analysis was done using GraphPad Prism V8 (Graphpad Software Inc., Version 8, La Jolla, CA, USA) and OriginLabs (OriginLab Corporation, Northampton, MA, USA). Significance was set at p < 0.05.

## Results

### Participants and disease characteristics

During the study period of July 2021 to October 2021, 10 ME/CFS patients and 10 age- and sex-matched HC participated in this project. All ME/CFS patients reported symptoms fulfilling the CCC case definition and no other fatigue-related illness that may account for their symptoms. Table 1 includes demographic data of the participants. The average age of participants was 44.10 ± 10.39 and 43.90 ± 10.71 for HC and ME/CFS patients, respectively. All participants who volunteered in this project were female except n=1. The average BMI of HC were within normal range (18.5–24.9) measuring at 23.68 ± 3.96. While the average BMI of ME/CFS patients were above normal range at 25.74 ± 5.49. There was a significant difference in employment status between HC and ME/CFS patients (p=0.009). Six of the included ME/CFS patients reported unemployment due to illness and/or disability.

**Table 1:** Participant demographics

	HC	ME/CFS	P-value
Age (years)	44.10 ± 10.39	43.9 ± 10.71	0.853
Gender n (%)			> 0.9999
Female	9 (90.0%)	9 (90.0%)	
Male	1 (10.0%)	1 (10.0%)	
BMI (kg/m^2^)	23.68 ± 3.96	25.74 ± 5.49	0.280
Employment Status			
Full Time	6 (60.0%)	2 (20.0%)	**0.0090**
Part Time	3 (30.0%)	1 (10.0%)	
Casual	1 (10.0%)	1 (10.0%)	
Unemployed	0 (0.0%)	0 (0.0%)	
Illness/disability	0 (0.0%)	6 (60.0%)	
Education			
Primary education	0 (0.0%)	0 (0.0%)	0.9985
High school	0 (0.0%)	1 (10.0%)	
Undergraduate	5 (50.0%)	3 (30.0%)	
Postgraduate/doctoral	2 (20.0%)	4 (40.0%)	
Other	3 (30.0%)	2 (20.0%)	

Values in bold are statistically significant

*HC* healthy controls, *ME* Myalgic encephalomyelitis, *CFS* chronic fatigue syndrome, *BMI* body mass index

The SF-36 and WHO DAS surveys were used to assess QoL in ME/CFS patients compared with HC. As reported in Table 2, means scores were significantly reduced in ME/CFS patients across all SF-36 domains compared with HC. Lowest SF-36 scores in ME/CFS patients were observed in limitations due to physical role (24.38 ± 19.19). Mean domain scores for the WHO DAS show a significant increase in disability in ME/CFS patients compared with HC. ME/CFS patients reported greatest difficulty in ability to maintain life activities (66.87 ± 24.66). All blood parameters were within normal range according to Queensland Health Pathology.

**Table 2:** Participant Quality of Life, disability scores and serology

	HC	ME/CFS	P-value
SF-36 (%)			
Physical functioning	94.0 ± 17.29	37.5 ± 30.93	** < 0.0001**
Physical role	95.0 ± 11.71	24.38 ± 19.19	** < 0.0001**
Pain	90.25 ± 20.83	40.0 ± 27.99	**0.003**
General Health	78.75 ± 11.69	29.59 ± 18.37	** < 0.0001**
Social functioning	98.75 ± 3.95	27.5 ± 26.22	** < 0.0001**
Emotional role	99.17 ± 2.63	69.99 ± 23.31	**0.002**
Emotional wellbeing	76.96 ± 11.04	39.17 ± 12.23	** < 0.0001**
WHO DAS (%)			
Communication & understanding	1.62 ± 2.09	45.83 ± 24.29	** < 0.0001**
Mobility	2.50 ± 6.35	56.0 ± 30.26	** < 0.0001**
Self-care	0.0 ± 0.0	33.13 ± 29.47	** < 0.0001**
Interpersonal relationships	1.25 ± 10.62	33.13 ± 31.6	**0.009**
Life activities	6.25 ± 10.62	66.87 ± 24.66	** < 0.0001**
Participation in Society	2.19 ± 3.62	57.49 ± 23.11	** < 0.0001**
Full blood count			
White Cell Count (× 10^9^/L)	5.97 ± 0.79	5.58 ± 0.84	0.247
Lymphocytes (× 10^9^/L)	2.02 ± 0.69	1.60 ± 0.42	0.315
Neutrophils (× 10^9^/L)	3.37 ± 0.69	3.29 ± 0.86	0.684
Monocytes (× 10^9^/L)	0.44 ± 0.10	0.41 ± 0.07	0.436
Eosinophils (× 10^9^/L)	0.14 ± 0.11	0.16 ± 0.09	0.579
Basophils (× 10^9^/L)	0.03 ± 0.01	0.04 ± 0.01	0.075
Platelets (× 10^9^/L)	250.40 ± 55.41	255.4 ± 31.49	0.684
Red Cell Count (× 10^12^/L)	4.47 ± 0.45	4.47 ± 0.34	0.912
Haematocrit	0.41 ± 0.04	0.41 ± 0.03	0.631
Haemoglobin (g/L)	134.40 ± 16.34	135.4 ± 11.71	0.853

Values in bold are statistically significant

*HC* healthy controls, *ME* Myalgic encephalomyelitis, *CFS* chronic fatigue syndrome, *SF-36* 36-item short form survey, *WHO* world health organization, *DAS* disability assessment schedule

All ME/CFS patients successfully completed the NCNED registry questionnaire. Data extracted from relevant questionnaire responses are presented Table 3. On average, patients were 26.7 years of age at the time of diagnosis and experienced symptoms of ME/CFS for 17.7 years. Eight of the ten ME/CFS patients (80.0%) included in this present study reported an infection prior to onset of symptoms. All ME/CFS patients (100%) reported experiencing key symptoms of ME/CFS including cognitive difficulties, body pain and sleep disturbances.

**Table 3:** ME/CFS symptom characteristics

Age of diagnosis (Years [Mean ± SD])		26.70 ± 12.75
Disease duration (Years [Mean ± SD])		17.70 ± 14.95
Infectious onset, n (%)		8 (80.0%)
Cognitive difficulties	Yes	10 (100%)
	No	0 (0.0%)
Pain	Yes	10 (100%)
	No	0 (0%)
Sleep disturbances	Yes	10 (100%)
	No	0 (0%)
Sensory disturbances	Yes	9 (90.0%)
	No	1 (10.0%)
Immune disturbances	Yes	9 (90.0%)
	No	1 (10.0%)
Gastrointestinal disturbances	Yes	9 (90.0%)
	No	1 (10.0%)
Cardiovascular disturbances	Yes	8 (80.0%)
	No	2 (20.0%)
Respiratory disturbances	Yes	8 (90.0%)
	No	2 (20.0%)
Thermostatic instability	Yes	10 (100%)
	No	0 (0%)
Urinary disturbances	Yes	5 (50.0%)
	No	5 (50.0%)

*ME* Myalgic encephalomyelitis, *CFS* chronic fatigue syndrome, *SD* standard deviation, *n* number

### Effect of PregS on calcium influx

**Fig. 1:**
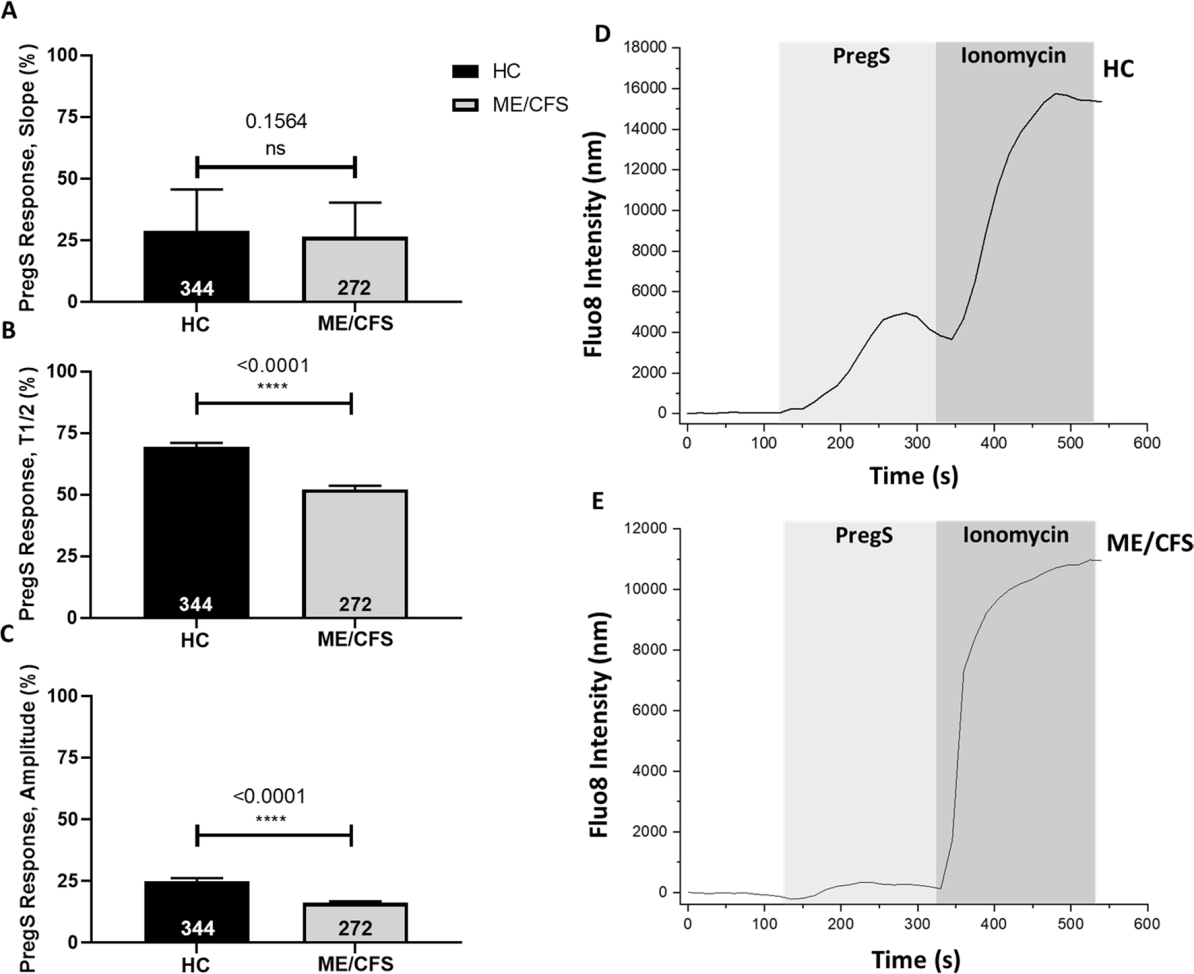
Stimulation of NK cells by 50 µM PregS at baseline prior to overnight stimulation with IL-2 and treatment with NTX. **A** Proportional mean data from Ca^2+^ imaging experiments comparing normalised slope of 50 µM PregS Ca^2+^ influx curve. **B** Proportional mean data from Ca^2+^ imaging experiments comparing normalised T1/2 (seconds) response of maximum 50 µM PregS Ca^2+^ response. **C** Proportional mean data from Ca^2+^ imaging experiments comparing normalised amplitude (nm) of maximum 50 µM PregS Ca^2+^ response. **D** Example time-course responses to 50 µM PregS and 1 µM Ionomycin in HC. **E** Example time-course responses to 50 µM PregS and 1 µM Ionomycin in ME/CFS patients. Data was collected from n = 10 HC and n = 10 ME/CFS patients. All Ca^2+^ influx measurements for PregS were normalised using Ionomycin response curves. Total number of cells following the removal of outliers are included within bar graphs. Data presented as mean ± SD. *HC* healthy controls, *ME* myalgic encephalomyelitis, *CFS* chronic fatigue syndrome, *T1/2* half-time, *nm* nanometres, *PregS*, pregnenolone sulfate, *SD* standard deviation, *ns* no significance

Ca^2+^ influx images were obtained from isolated human NK cells prior to overnight incubation with IL-2 and NTX (Fig. 1). TRPM3-dependent Ca^2+^ influx was stimulated by 50 µM of PregS. While there was no significant difference reported for the slope of the Ca^2+^ influx curve between groups, the T1/2 response (p < 0.0001) and amplitude (p < 0.0001) were significantly reduced in ME/CFS patients compared with HC. The inhibition of 50 µM PregS by ononetin was also determined at baseline. Ononetin at a concentration of 4 µM effectively blocked PregS stimulation of TRPM3, however there was no statistical significance between groups (Additional file 1: Figure S3.1).

### Effect of PregS on calcium influx after NTX treatment

**Fig. 2:**
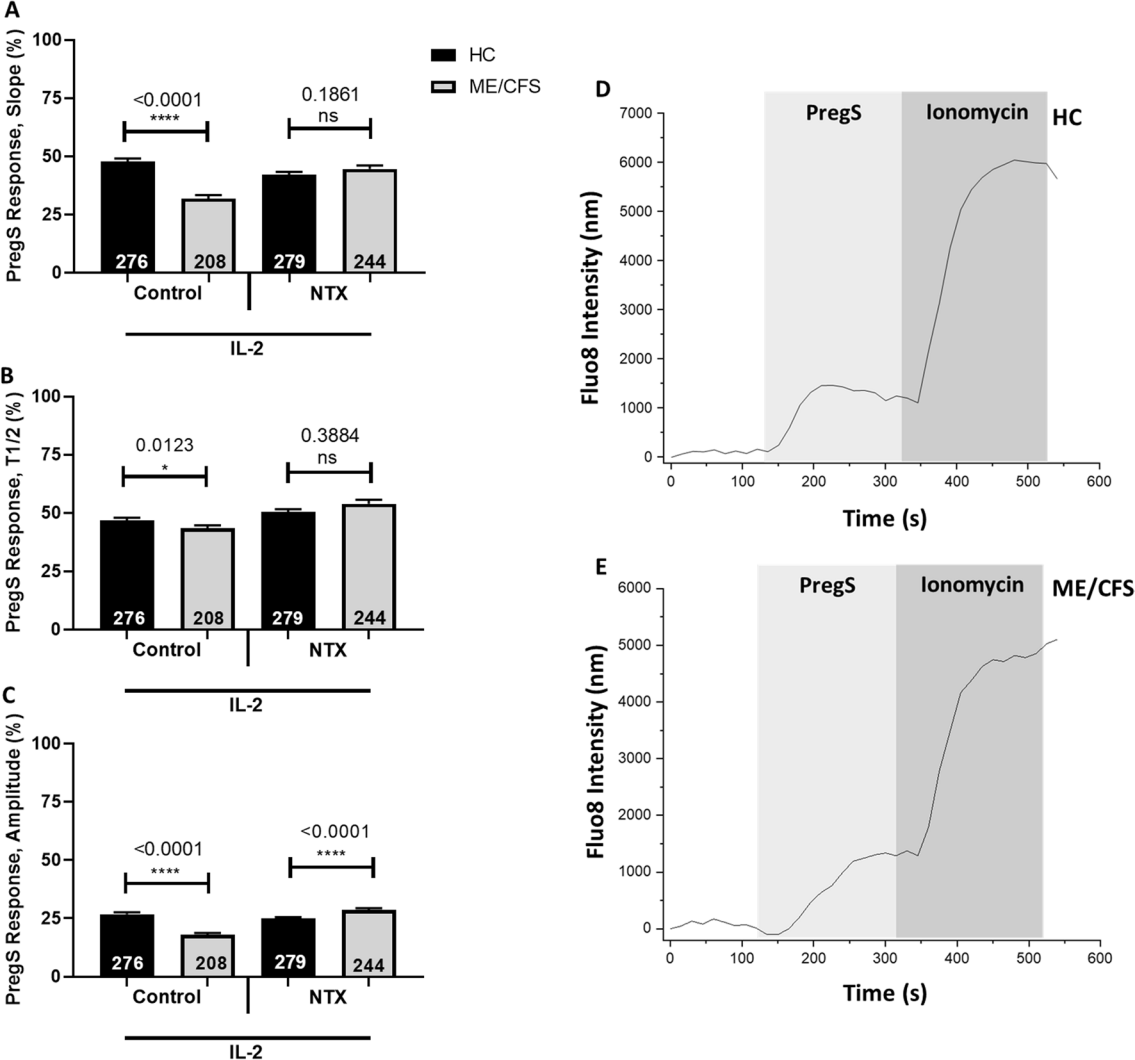
Stimulation of NK cells by 50 µM PregS following overnight stimulation with IL-2 and treatment with NTX. **A** Proportional mean data from Ca^2+^ imaging experiments comparing normalised slope of 50 µM PregS Ca^2+^ influx curve. **B** Proportional mean data from Ca^2+^ imaging experiments comparing normalised T1/2 (seconds) response of maximum 50 µM PregS Ca^2+^ response. **C** Proportional mean data from Ca^2+^ imaging experiments comparing normalised amplitude (nm) of maximum 50 µM PregS Ca^2+^ response. **D** Example time-course responses to 50 µM PregS and 1 µM Ionomycin in HC. **E** Example time-course responses to 50 µM PregS and 1 µM Ionomycin in ME/CFS patients. Data was collected from n = 10 HC and n = 10 ME/CFS patients. All Ca^2+^ influx measurements for PregS were normalised using Ionomycin response curves. Total number of cells following the removal of outliers are included within bar graphs. Data presented as mean ± SD. *HC* healthy controls, *ME* myalgic encephalomyelitis, *CFS* chronic fatigue syndrome, *T1/2* half-time, *nm* nanometres, *PregS*, pregnenolone sulfate, *SD* standard deviation, *ns* no significance

Human NK cells were incubated overnight supplemented with IL-2 with and without NTX (Fig. 2). Post-24 h, Ca^2+^ influx images were obtained from both control IL-2 stimulated cells and NTX treated cells. There was a significant reduction in slope (p < 0.0001), T1/2 response (p = 0.0123) and amplitude (p < 0.0001) in ME/CFS patients compared with HC. There was no significant difference for NTX treated cells between groups for slope and T1/2 response. A significant increase in amplitude (p < 0.0001) was reported in ME/CFS patients compared with HC following NTX treatment. The inhibition of 50 µM PregS by ononetin was also determined following stimulation of NK cells with IL-2 and treatment using NTX. Ononetin at a concentration of 4 µM effectively blocked PregS stimulation of TRPM3, however there was no statistical significance between groups (Additional file 1: Figure S3.2).

## Discussion

We report, for the first time the significant reduction in Ca^2+^ influx via TRPM3 in NK cells in ME/CFS patients compared with HC using Ca^2+^ imaging technique. This current investigation provides novel findings for the rate, or speed, and insight into the maximum TRPM3-dependent Ca^2+^ influx response in ME/CFS patients. Previous investigations have demonstrated TRPM3 channel dysfunction in isolated NK cells of ME/CFS patients compared with HC using the whole-cell patch clamp technique [10, 11]. While patch-clamp is regarded as a gold standard technique for ion channel research, TRPM3-dependent Ca^2+^ influx was not clear in our previous studies due to the small inward currents activated by PregS. Therefore, our data supports the characterisation of Ca^2+^ influx and TRPM3 activity for the pathomechanism of ME/CFS and the role for NTX as a potential intervention. The combination of electrophysiology and Ca^2+^ imaging protocols provides a comprehensive and complete analysis of TRPM3-dependent Ca^2+^ changes in NK cells of ME/CFS patients.

This current investigation reports a significant reduction in the T1/2 and amplitude of maximum TRPM3-dependent Ca^2+^ responses in ME/CFS patients compared with HC at baseline. The reduction in TRPM3-dependent Ca^2+^ influx in ME/CFS patients compared with HC validates previous research which demonstrate significant loss in TRPM3 ion channel function [10, 11, 25]. Alterations in the profile of Ca^2+^ influx are strongly associated with significant physiological consequences. Changes in Ca^2+^ influx profiles play a critical role in determining the magnitude of Ca^2+^-dependent responses and are associated with physiological consequences [37]. It is hypothesised that TRPM3 ion channel dysfunction results in reduced Ca^2+^ influx in NK cells which has negative consequences on cytotoxic function in ME/CFS patients. Therefore, simultaneous reductions in amplitude and rate may augment effects of reduced Ca^2+^ concentration on cellular function and further research into Ca^2+^ mobilisation may elucidate the pathomechanism of ME/CFS.

Subsequently, the effect of IL-2 alone on Ca^2+^ influx was determined. The culturing of NK cells with the addition of IL-2 supports viability, preactivation, proliferation and development of cells; however, as IL-2 primes NK cells for activation this condition was used as a control for NTX treated cells [38]. The amplitude and T1/2 response were significantly reduced in IL-2 stimulated NK cells from ME/CFS patients compared with HC. Additionally, a significant reduction was reported for slope of Ca^2+^ influx in ME/CFS patients compared with HC. It is interesting that significance was reported for slope of Ca^2+^ influx following IL-2 stimulation, but not at baseline. Ca^2+^ measurements differed between baseline and IL-2 stimulation conditions. Stimulation of NK cells with IL-2 is known to enhance NK cell cytotoxic function through Ca^2+^-dependent pathways [39, 40]. In a recent publication, the authors suggested a crosstalk between TRPM3- and IL-2-dependent cellular pathways leading to enhanced NK cell function in vitro [41]. It is therefore likely that changes in slope reported between baseline and IL-2 stimulation that are reflected in HC indicate that pathways involved in TRPM3 and IL-2 signalling may promote Ca^2+^ mobilisation in NK cells. The consistent reduction in amplitude and T1/2 of response reported in this current investigation suggests that TRPM3 dysfunction reported in ME/CFS patients impairs sufficient Ca^2+^ entry in NK cells. Changes in ion channel function may be a consequence of an unstable open channel [42] and channel stability may be a component to consider in future research.

Ca^2+^ has a critical and beneficial role in promoting the activation of NK cell function as previous investigations using Ca^2+^ blockers have led to a significant reduction in NK cell cytotoxicity [43, 44]. The coupling between receptor and ligand initiates a Ca^2+^-dependent cascade that relies on sustained and long-term influx of the cation. Ca^2+^ facilitates the phosphorylation and activation of protein kinases such as Ras, P38, phosphatidylinositol 4,5-bisphosphate 3-kinase (PI3K) and mitogen activation protein kinases (MAPK) by enabling protein–protein and protein-phosphatase interactions [45–47]. The significant reduction in phosphorylation of protein kinases has been reported in activated NK cells of ME/CFS patients [48]. Therefore, changes in Ca^2+^ can either enhance, or in the case of ME/CFS, impair protein kinase phosphorylation, thus impacting functional outcomes such as cytokine production and cytotoxicity. Moreover, phosphatidylinositol 4,5-bisphosphate (PIP_2_) is a critical component of numerous signalling pathways including PI3K signalling and Ca^2+^ mobilisation. The function of TRPM3 also relies on the presence and binding of PIP_2_ [49]. A recent publication reported a potential association between TRPM3 dysfunction and the involvement of PIP_2_ in the pathomechanism of ME/CFS [41]. Therefore, TRPM3 dysfunction may contribute to ME/CFS pathomechanism due to consequences of impaired Ca^2+^ signalling, including impeding Ca^2+^-dependent cellular pathways that result in impaired NK cells function that is consistently reported in literature.

Under homeostatic conditions, the activation of TRPM3 leads to an increase in cytosolic Ca^2+^ that is important for regulating numerous biological processes including but not limited to temperature and pain sensation, insulin secretion and vascular smooth muscle contraction [50]. Furthermore, ion channels are located on different organelles throughout cells, and mitochondrial dysfunction reported in ME/CFS patients may be attributable to ion channel dysfunction, namely TRPM3 [51]. In NK cells, store-operated Ca^2+^ entry (SOCE) is a primary form of Ca^2+^ regulation [52]. However, it is possible that TRPM3 monitors intracellular Ca^2+^ levels in lymphocytes in addition to SOCE, as observed in oligodendrocytes [53, 54]. This current investigation did not aim to assess the activity of SOCE and therefore further investigation on the relationship between TRPM3 and SOCE in NK cells is proposed. It is important to outline that within the N-terminal of TRPM3 there are two calmodulin (CaM) binding sites. CaM senses changes in [Ca^2+^]_I_ to either up- or down-regulate TRPM3 activity [55, 56]. The interaction of CaM and TRPM3 suggests strong activity in Ca^2+^ sensing that may be augmented by Ca^2+^ store depletion or by stimulation of GPCRs [57]. The activity of CaM in TRPM3 function has not been investigated in ME/CFS patients but provides an interesting target for future research to determine any problems in TRPM3-dependent Ca^2+^ homeostasis.

Currently, there is no universal treatment to improve symptoms of ME/CFS. However, new technologies may provide future avenues to further assist in characterising TRPM3 dysfunction and impaired Ca^2+^ mobilisation in ME/CFS research. Recent electrophysiology investigations have reported the restoration of TRPM3 function following in vitro treatment of isolated NK cells with NTX [25]. In this current investigation we report the restoration of TRPM3-dependent Ca^2+^ influx following in vitro treatment of isolated NK cells overnight with NTX. The cellular mechanism involves the inhibition of µOR which would otherwise inhibit the activation of TRPM3 channels [28, 58]. Therefore, by negating the inhibitory action of µOR, it is hypothesised that this restores TRPM3 function, thus reinstating TRPM3-dependent Ca^2+^ influx in NK cells from ME/CFS patients.

The expression of µOR has been reported in lymphocytes where they are important in the inflammatory pain response [59]. The activation of µOR by certain endogenous and synthetic opioids leads to immunosuppression [60, 61, 61, 62]. Administration of the synthetic opioid, morphine, has resulted in a significant reduction in NK cell cytotoxic activity in rats, mice and humans [63–65]. Interestingly, NTX inhibits the suppressive activity of morphine on NK cells through the µOR [66]. Research has reported that ME/CFS patients secrete insufficient opioid peptides that regulate pain and release of cytokines [31]. Beta-endorphins (β-EP) are an endogenous opioid neuropeptide produced by many cell types including lymphocytes. The release of β-EP from lymphocytes are regulated by the activity of µOR resulting in changes to cell proliferation and immune function [67]. Interestingly, within a specific dosage window of 1–5 mg/day, it is reported that LDN increases β-EP release in NK cells resulting in increased cytotoxic activity [68, 69]. An investigation by Conti and colleagues found significantly reduced β-EP in ME/CFS patients reflecting chronic immune activation [69]. Therefore, targeting OR, with NTX, on immune cells may resolve NK cell dysfunction through ion channel regulation such as the indirect effects seen with TRPM3. Future research may aim to investigate NK cell cytotoxicity in ME/CFS patients following in vitro treatment with NTX.

TRPM3 function has previously been assessed in a group of ME/CFS patients who routinely administer LDN daily [31]. ME/CFS patients who take LDN yielded similar TRPM3 function to HC and no significant differences between groups were observed [31]. Medical data from a cohort of 218 ME/CFS patients administering LDN reported a positive treatment response in 73.9% of responders [24]. In a separate investigation, self-reported retrospective comparison of ME/CFS symptoms before and after LDN commencement suggested an improvement in cognitive symptoms and immune disturbances [31]. Moreover, studies with fibromyalgia patients have found that LDN significantly reduced pain, fatigue, sleep disturbances, headaches and gastrointestinal issues [70]. Collectively, these results suggest there is potential therapeutic benefit of LDN to treat TRPM3 dysfunction in ME/CFS patients.

## Conclusion

ME/CFS is a serious and chronic condition that affects multiple organ systems of the human body. TRPM3 dysfunction has been consistently found in NK cells of ME/CFS, with consequential implications for Ca^2+^ signalling and cell function. TRPM3-dependent Ca^2+^ influx was significantly reduced in NK cells of ME/CFS patients compared with HC. In this current investigation the treatment of isolated NK cells with NTX restored TRPM3-dependent Ca^2+^ influx in ME/CFS patients to the extent that Ca^2+^ influx measurements were either similar to HC or significantly increased. This investigation provides additional evidence for the potential therapeutic benefit of NTX for ME/CFS.

## Supplementary Information

**Additional file 1.** Supplementary figures.

## Data Availability

Datasets analysed and/or generated during the current study are not publicly available due to confidentiality agreements but are available from the corresponding author upon reasonable request.

## Abbreviations

β-EP: Beta endorphins
µ: Mu
Ca^2^^+^: Calcium
[Ca^2^^+^]_I_: Intracellular calcium concentration
CaM: Calmodulin
CCC: Canadian Consensus Criteria
CFS: Chronic fatigue syndrome
CNS: Central nervous system
CRAC: Calcium release activated channel
DAS: Disability assessment schedule
DRG: Dorsal root ganglia
EDTA: Ethylenediaminetetraacetic acid
ERK: Extracellular-signal-release kinase
GPCR: G protein coupled receptors
HC: Healthy controls
ICC: International Consensus Criteria
IL-2: Interleukin 2
IP_3_: Inositol 1,3 bisphosphate
LDN: Low dose naltrexone
MAPK: Mitogen activated protein kinase
ME: Myalgic encephalomyelitis
NK: Natural killer
NTX: Naltrexone hydrochloride
OR: Opioid receptor
PBMC: Peripheral blood mononuclear cells
PI3K: Phosphatidylinositol 4,5-bisphosphate 3-kinase
PIP_2_: Phosphatidylinositol 4,5-bisphosphate
PLC: Protein lipase C
PregS: Pregnenolone sulfate
QoL: Quality of life
SD: Standard deviation
SF-36: 36 Item short form healthy survey
SOCE: Store operated calcium entry
T1/2: Half-time
TRPM3: Transient receptor potential melastatin 3
WHO: World Health Organization
