# Supplementary material for: Impaired TRPM3-dependent calcium influx and restoration using Naltrexone in natural killer cells of myalgic encephalomyelitis/chronic fatigue syndrome patients
Source: J Transl Med. 2022 Feb 16;20:94. doi: 10.1186/s12967-022-03297-8 (PMC8848670; doi:10.1186/s12967-022-03297-8)
Supplement: Supplementary file 1 — Additional file 1. Supplementary figures. [file 12967_2022_3297_MOESM1_ESM.docx]

# **Additional material**

**Additional file 1: Figure S1: NK Cell Purity**

**A**

**B**

**C**


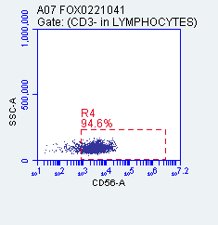

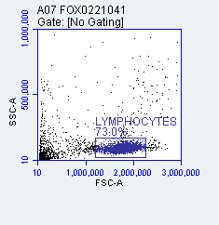

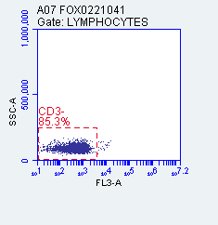


Additional file 1: Figure S1: Gating strategy: natural killer (NK) cells were stained with CD3 APH-H7 (5µl/test) and CD56 BV650 (20µl/test) monoclonal antibodies (Becton Dickinson [BD] Biosciences, San Jose, CA, USA). Cells were acquired at 10,000 events using the Accuri C6 flow cytometer (BD Biosciences, San Diego, CA, USA). Gating strategy is as follows: (A) lymphocytes were gated based of SSC and FSC. (B) CD3 negative population was gated from selected lymphocyte population. Gating was determined using isotype controls. (C) NK cell purity was determined based on CD56 positive cells using the CD3 negative population.

**Additional file 1: Figure S2: NK Cell Purity**

Additional file 1: Figure S2: Bar graphs representing natural killer (NK) cell purity (%) determined using flow cytometry methods. HC NK cell purity (%) was 90.02 ± 4.79 and ME/CFS NK cell purity (%) was 84.68 ± 9.38. Data presented as mean ± SD. Abbreviation: HC, healthy controls; ME/CFS, myalgic encephalomyelitis/chronic fatigue syndrome; SD, standard deviation.

**Additional file 1: Figure S3: Ononetin Protocol and Results**

Due to the natural decline in fluorescence following PregS stimulation of TRPM3, the action of ononetin was determined in a separate protocol. In order to desensitize TRPM3 channels, baseline Ca^2+^ was imaged for 3 minutes in the presence of 4µM of ononetin followed by ononetin 4µM + PregS 50µM, followed by PregS 50µM and lastly ionomycin 1µM. Similarly, to the analysis of PregS as seen in the manuscript, three methods of analysing Ca^2+^ influx was used: slope, T1/2 and amplitude. These measurements were then normalised against the response to ionomycin.

PregS inhibition by ononetin was determined at baseline prior to (Additional file 1: Figure S3.1) and following (Additional file 1: Figure S3.2) stimulation of isolated NK cells with IL-2 and treatment usingNTX. Ononetin at a concentration of 4µM effectively blocked PregS stimulation of TRPM3. There was no statistical significance between groups across all conditions.


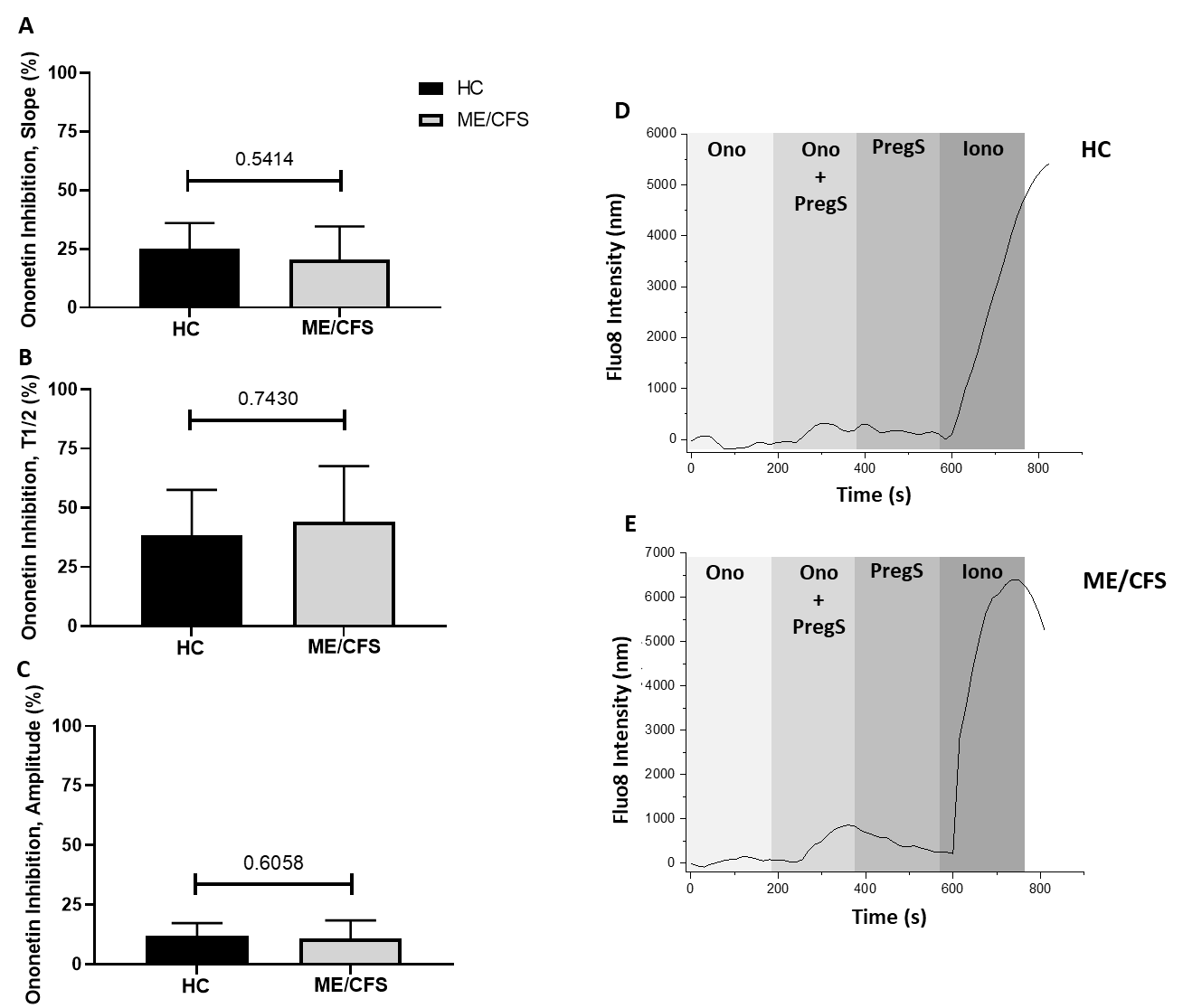


Additional file 1: Figure S3.1: Baseline ononetin Ca^2+^ measurements for HC and ME/CFS patients prior to overnight stimulation with IL-2 and treatment with NTX. (A) Mean data from Ca^2+^ imaging experiments comparing normalised slope values of 4µm ononetin + 50µM PregS Ca^2+^ responses. (B) Mean data from Ca^2+^ imaging experiments comparing normalised T1/2 (s) values of 4µm ononetin + 50µM PregS Ca^2+^ responses. (C Mean data from Ca^2+^ imaging experiments comparing normalised amplitude (nm) values of 4µm ononetin + 50µM PregS Ca^2+^ responses. (D) Example time-response curves for HC. (E) Example time-response curves for ME/CFS patients. Data represented mean ± SD. Data presented as mean ± SD. Abbreviations: HC, healthy controls; ME, myalgic encephalomyelitis; CFS, chronic fatigue syndrome; T1/2, half-time; nm, nanometres; PregS, pregnenolone sulfate; SD, standard deviation.


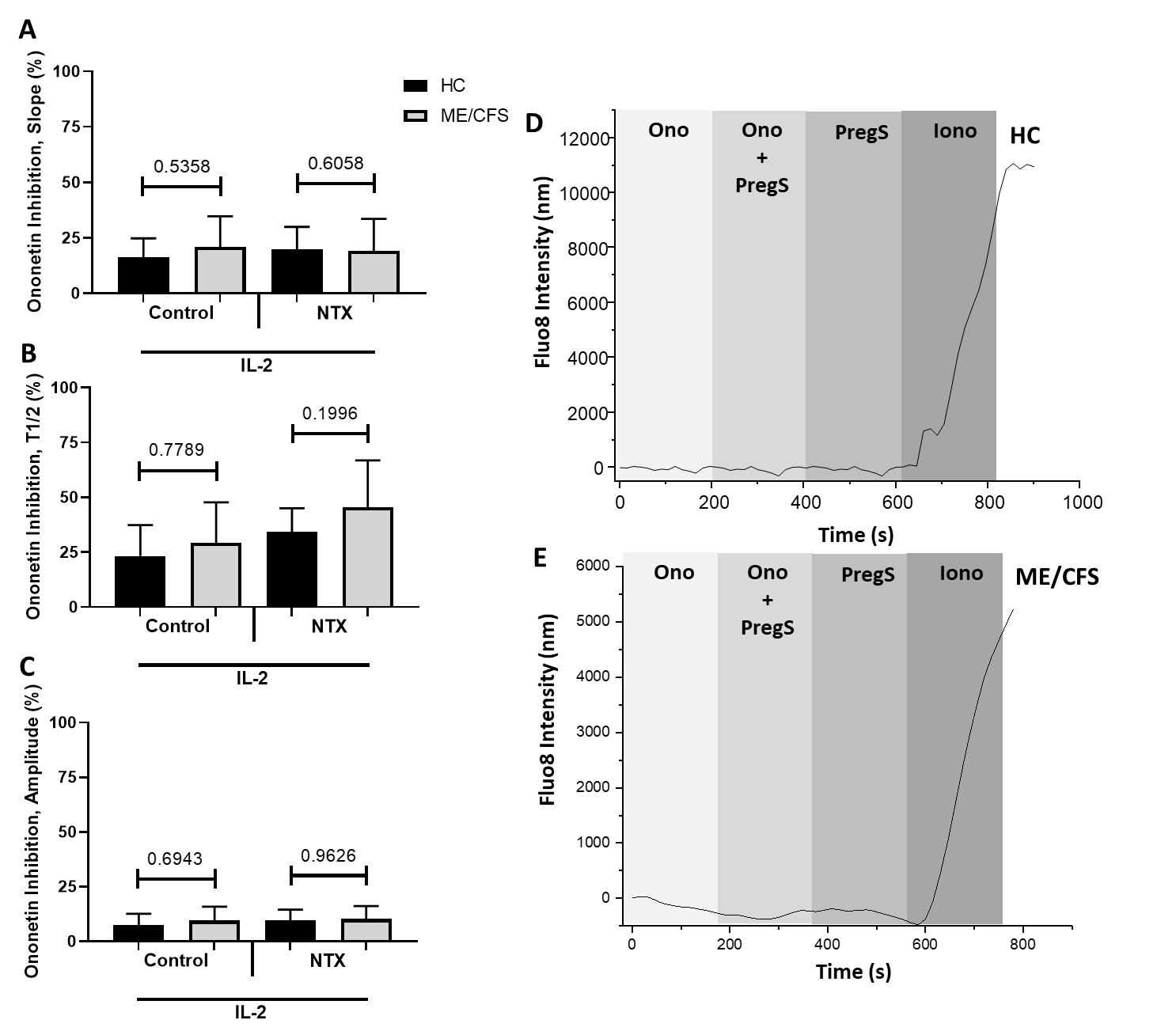


Additional file 1: Figure S3.2: Stimulation of NK cells by 50µM PregS following overnight stimulation with IL-2 and treatment with NTX. (A) Mean data from Ca^2+^ imaging experiments comparing normalised slope values of 4µm ononetin + 50µM PregS Ca^2+^ responses. (B) Mean data from Ca^2+^ imaging experiments comparing normalised T1/2 (s) values of 4µm ononetin + 50µM PregS Ca^2+^ responses. (C Mean data from Ca^2+^ imaging experiments comparing normalised amplitude (nm) values of 4µm ononetin + 50µM PregS Ca^2+^ responses. (D) Example time-response curves for HC. (E) Example time-response curves for ME/CFS patients. Data represented mean ± SD. Data presented as mean ± SD. Abbreviations: HC, healthy controls; ME, myalgic encephalomyelitis; CFS, chronic fatigue syndrome; T1/2, half-time; nm, nanometres; PregS, pregnenolone sulfate; SD, standard deviation.

**Additional file 1: Figure S4: PregS and Ononetin Dose Response**

NK cells isolated from HC (n=3) were used to determine the EC_50_ (half maximal effective concentration) of PregS using Ca^2+^ imaging protocols as outlined in the main body of the manuscript (Additional file 1: Figure S4.1). The highest EC50 (50µM) was used in order to reduce any variations in response from cell to cell.

**
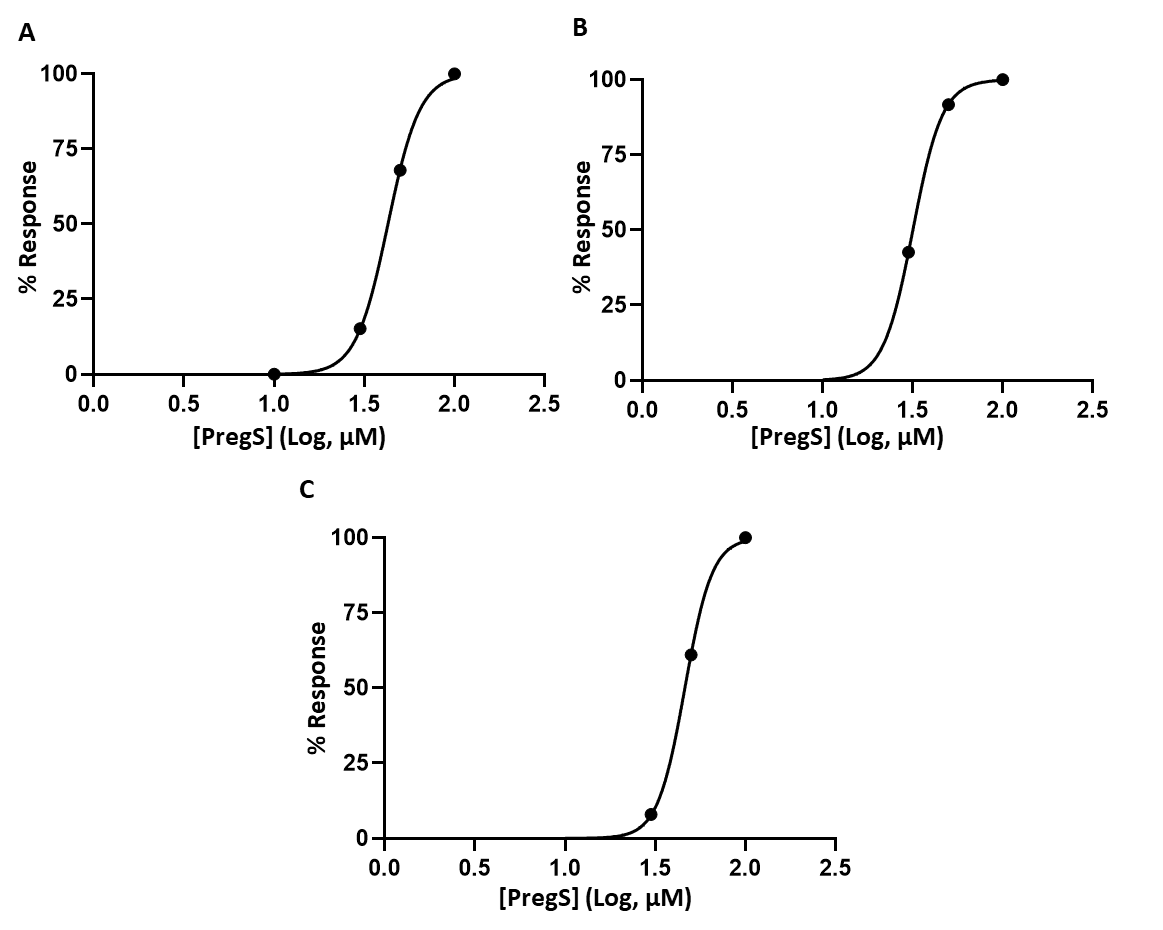
**

Additional file 1: Figure S4.1: EC_50_ of PregS assessed in NK cells subjected to Ca^2+^ imaging experiments. (A) dose response curve constructed from mean data of normalised slope values, EC50=49.73µM. (B) dose response curve constructed from mean data of normalised T1/2 values, EC50=31.74µM.

NK cells isolated from HC (n=5) were used to determine the IC_50_ (half maximal inhibitory concentration) of ononetin using Ca^2+^ imaging protocols as outlined in the main body of the manuscript (Additional file 1: Figure S4.2).


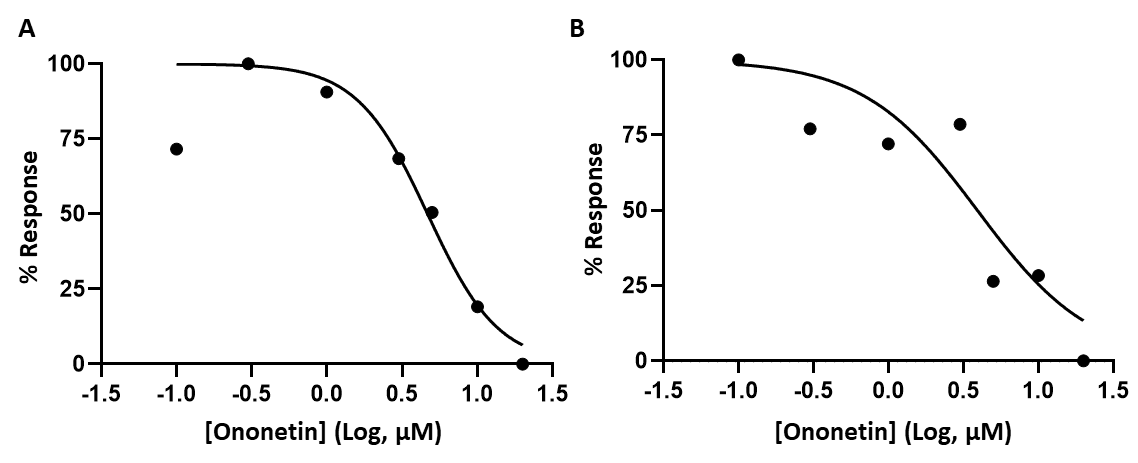


Additional file 1: Figure S4.2: IC_50_ of ononetin assessed in NK cells subjected to Ca^2+^ imaging experiments. Different doses of ononetin were used to determine inhibition of 50µM of PregS (A) dose response curve constructed from mean data of normalised slope values, IC50 = 4.67µM. (B) dose response curve constructed from mean data of normalised T1/2 values, IC50 = 3.92µM.
